# Undoing SUMO aids polyQ AR

**DOI:** 10.18632/oncotarget.5862

**Published:** 2015-09-28

**Authors:** Andrew P. Lieberman, Jorge A. Iniguez-Lluhi

**Affiliations:** Department of Pathology, University of Michigan, Ann Arbor, Michigan, USA

**Keywords:** androgen receptor, SUMO, polyglutamine, spinal and bulbar muscular atrophy

Of the age-dependent protein aggregation disorders associated with neurodegeneration, diseases caused by polyglutamine tract expansions have attracted particular attention. These inherited disorders are caused by CAG microsatellite expansions in the coding regions of disease-causing genes. These alterations lead to degeneration that tends to present in mid-life, progress slowly, and exhibit toxicity that is intimately associated with misfolding of the mutant protein. This proteotoxic gain-of-function is a shared common feature that contributes to many disease manifestations.

Variation in the length of CAG/glutamine tracts is also known to influence the normal function of the affected proteins. This has been well studied in the case of the androgen receptor (AR), where variations in tract length that occur within the normal population revealed that length is inversely associated with function of the receptor as a ligand activated transcription factor. Further pathological expansion of the AR's CAG repeat causes spinal and bulbar muscular atrophy (SBMA), an untreatable and progressive degenerative disorder of the neuromuscular system characterized by skeletal muscle weakness and atrophy, and loss of motor neurons in the spinal cord and brainstem. This disease is X-linked, and affected men often show signs of partial androgen insensitivity including gynecomastia, testicular atrophy and decreased fertility. These clinical features correlate with diminished intrinsic transcriptional regulatory activity of the polyglutamine expanded AR (polyQ AR).

Several general principles guide our understanding of SBMA pathogenesis. Ligand binding to the polyQ AR promotes nuclear translocation and protein unfolding. Both of these steps are required for pathogenesis hence the occurrence of disease only in men. In SBMA, as in other CAG repeat disorders, the mutant protein disrupts multiple downstream pathways and toxicity likely results from the cumulative effects of altering a diverse array of cellular processes including transcription, RNA processing, axonal transport, and mitochondrial function. Notably, recent studies have established that skeletal muscle is an important site of toxicity and an attractive therapeutic target. SBMA patient muscle shows both denervation and myofiber degeneration, genetic deletion of a floxed polyQ AR allele only in skeletal muscle rescues disease in BAC transgenic mice [1], and treatment of knock-in mice with subcutaneously administered antisense oligonucleotides diminishes polyQ AR expression in the periphery but not CNS and rescues disease [2].

While SBMA patients exhibit signs of partial androgen insensitivity, the extent to which loss of AR function contributes to pathogenesis has been poorly understood. A major challenge has been the difficulty in dissociating effects of polyQ AR proteotoxicity from impaired intrinsic transcriptional function. To accomplish this, recent work mitigated the transcriptional deficits of the polyQ AR by relieving the inhibitory effect of AR posttranslational modification by small ubiquitin-like modifier (SUMO) [3]. SUMO proteins share with ubiquitin a common structural motif and are reversibly conjugated to target proteins at lysine residues. AR was one of the first proteins shown to be SUMOylated and this modification both occurs at, and mediates the function of two short amino acid motifs found in the N-terminal region of the receptor. These motifs inhibit the transcriptional activity of AR in a unique promoter context dependent manner [4]. Although the steady-state stoichiometry of AR SUMOylation is low, SUMO exerts its strong inhibitory effects by causing redistribution of AR away from chromatin through the recruitment of distinct partner proteins [5]. Relief from SUMO-mediated inhibition through mutation of the acceptor lysines to arginines abrogated polyQ AR SUMOylation *in vitro* [3, 6] and potentiated polyQ AR function as a ligand activated transcriptional regulator both *in vitro* and in gene targeted mice [3]. Moreover, disrupting polyQ AR SUMOylation rescued exercise endurance, type I muscle fiber atrophy and survival, thereby demonstrating a significant amelioration of disease. This phenotypic rescue in mice was hormone-dependent, indicating that it was mediated by activation of the mutant receptor [3].

This work demonstrates beneficial effects of enhancing polyQ AR transcriptional function in a mouse model of SBMA. Anabolic support of skeletal muscle by AR is well established, and promoting this function may ameliorate aspects of the disease. These findings are also notable because previous work in a cellular model indicated that acute elevations of AR SUMOylation can mitigate the ligand dependent aggregation of polyQ AR [6]. Thus, it appears that in the chronic context of the disease model, enhancement of AR activity due to loss of SUMOylation exerts a beneficial effect despite the potential loss of acute attenuation of aggregation. Phenotype rescue by disrupting polyQ AR SUMOylation may involve concurrent mechanisms in addition to enhanced AR activity. It is possible that altered receptor interactions in the absence of SUMOylation leads to the formation of less toxic species or to indirect effects on protein degradation pathways [7]. Future work should define the relative contributions of these alternative possibilities and further explore the potential for targeting the SUMOylation pathway for therapeutic benefit in SBMA.
